# Foreign Medical and Physical Science and Literature

**Published:** 1818-12

**Authors:** 


### FOREIGN MEDICAL AND PHYSICAL SCIENCE AND LITERATURE.

### *Transactions of the Physico-Medical Society of New York.* Vol. I.

(Concluded from our last Number.)

### *Reflections on the Pulsation in Epigastrio, with an Enquiry into its Causes;* by VALENTINE MOTT, M.D.

THIS Essay is the result of extensive researches respecting an obscure, but very interesting series of complaints; and, although Dr. MOTT has not adduced many new facts of great importance respecting them, yet his judicious arrangement and reflections on those we are already possessed of will serve to illustrate the observations of LIENTAUD, BAILLIE, PORTAL, ALBERS, and BURNS, and facilitate the labours of future enquirers.

Pulsation in the epigastrium, he considers, may arise from the following causes:—

1. Aneurism of the aorta, cœliac, or superior mesenteric artery.
2. Enlargement or disease of the pancreas.
3. Schirrus of the stomach, and particularly its lower or pyloric orifice.
4. Tumors at the root of the mesentery.
5. Nervous irritation.
6. Enlargement of the vena cava inferior.
7. Increased solidity of the lungs.
8. Enlargement of the heart, particularly a dilatation of its right side.
9. Adhesion of the pericardium to the heart.

Each of the above causes are verified by facts which have occurred to the observation of the author, or reference to those related by other writers. We shall give as extensive an abstract of them as our limits will permit.

1. Aneurism of the aorta, it is sufficiently evident, may occasion pulsation in the epigastrium, but it does not necessarily arise from that disease; many cases of aneurism of that artery have proved fatal, without ever having been suspected during life.*

Aneurism of the aorta about the cœliac and superior mesenteric arteries, is of less frequent occurrence than at other parts of the aortic trunk. Most of the symptoms which have been attributed

---

* HODGSON, p. 88.

to that as a cause arise from some other disease. "When I now," says Mr. A. Burns, "meet with a person having a pulsation in the epigastrium, I almost immediately decide, in my own mind, that the cœliac artery is healthy. I have met with above twenty instances of pulsating tumors in the region of the cœliac artery, and many of these were, by myself and others, supposed to be cœliac aneurisms; and yet it turned out, on dissection, that not one of all of them had originated from a diseased state of that or any other artery." Page 59, 60. In another place this author remarks, that the only case of cœliac aneurism he ever saw was not even suspected during life.

If the pulsation in the epigastrium is strong, we may infer, according to the above-mentioned author, that it is not aneurismal.

The perfect correspondence between the pulsation of the epigastrium and the action of the heart, is a circumstance strongly diagnostic of the aneurismal state of the aorta, cœliac, or superior mesenteric artery.

2. Disease of the *pancreas* is usually accompanied with pulsation in the epigastrium; indeed so generally, that Dr. Sewell considered it as an invariable pathognomonic symptom. It is noticed by Professor Warren, of Boston, and also by Mr. A. Burns. The pulsation may arise either from an interruption to the free circulation of blood through the hardened viscus, or, what is more probable, from the pressure of the enlarged and hardened pancreas upon the aorta, thereby intercepting the free passage of blood through it, by which it accumulates, and distends the trunk of the artery above into a more or less of an aneurismal state.

3. The situation and connexions of the right extremity of the stomach makes it less frequently a cause of pulsation, when diseased, than a schirrous condition of the pancreas. It has, however, been mentioned as a cause of it by Baillie,* Burns,† Monro,‡ and Francis.§

Like the schirrus of the pancreas, the obstruction to the free passage of blood through the hardened part of the stomach may give such an impetus in the surrounding vessels as to produce the same sensation. May not the tumors‖ of various sizes and consistence, which are occasionally found in the stomach, growing from the villous coat, give rise to the same symptoms?

4. The lacteal and lymphatic glands, about the root and in the duplicatures of the mesentery, and in the lumbar region, are sometimes much enlarged. These enlargements frequently produce a pulsatory motion in the abdomen: this may be in the lower part of the epigastric or umbilical regions, and is sometimes

---

* Morbid Anatomy. † Diseases of the Heart. ‡ Morbid Anatomy. § Trans. Lit. and Phil. Soc. of New-York, vol. i.

‖ Vide Fab. de Hildan, Bonetus, Ruysch, Morgagni, Lieutaud, Haller, Baillie, Monro, and particularly Portal's *Com. d'Anatomie Medic.* tom. v. p. 193, 198.

isochronous with the pulsation in the arteries of the extremities. A case of this kind is related by Dr. Albers, of Bremen, in the Edinburgh Medical and Surgical Journal, vol. iii.

The mechanical obstruction which these tumors occasion to the transmission of blood in the trunks or branches of the surrounding arteries will explain, as in the former affections, the pulsation in the epigastric or umbilical region.

5. That a pulsatory motion in the epigastic region should occur, unaccompanied with disease of any of the surrounding organs, is a curious and an interesting fact. Habits that are preternaturally susceptible of impressions—those whose nervous systems are deranged, are more especially the subjects of this distressing, and, for the most part, alarming affection. In hypochondriacal men and hysterical women, this pulsation often occasions the greatest distress. As the stomach is an organ possessing the most important and extensive intercourse with the various parts of the system in health; so, in a state of disease, do many morbid associations assemble about the præcordia. That nervous irritation should here be concentrated, and develop itself in the form of pulsation, is not more extraordinary than the phenomenon of blushing. The sudden manner of its attack is a circumstance which strongly favours this opinion. This forms a striking and diagnostic distinction between it and an aneurism of the aorta or surrounding branches. Aneurisms, for the most part, are of slow progress,—the pulsation continues to become stronger, and the tumor more apparent: but the pulsation from nervous irritation is suddenly induced,* remains with very little variation for many years, and perhaps for life. Dr. Baillie relates, that he saw a man who had been labouring under this pulsation for twenty-five years, when he consulted him: when it first attacked him, he had taken the opinion of Sir Cæsar Hawkins, Mr. Bromfield, and Dr. Hunter: the two first told him, it was an aneurism of the aorta; but Dr. Hunter saw so much peculiarity about it, that he ingenuously said he did not know what it was.†

This affection most commonly occurs about the middle period of life. It varies at different times of the day, and is often more strong in the afternoon and evening than in the morning. A lady, who had had several children, found this pulsation so certain a sign of pregnancy, that she confided in it in preference to all others. It was so violent at times, that her husband asserted it could be distinctly heard. It usually left her after the third month.‡

6. An instance of pulsation in the epigastrium is related by Senac, in which, on examination after death, the vena cava was found dilated and as large as a man's arm. "We have already," says Mr. Allan Burns, "seen some cases in which this had taken place; and that the pulsation is occasionally rendered more dis-

---

* Vide Dr. Albers' Paper, loc. cit.

† Dr. Baillie's Paper, Trans. Coll. Phys. London, vol. 4.

‡ See Dr. Albers' Paper, loc. cit.

tinct by the veins being at the same time dilated." Dr. Pemberton, in his Treatise on the Diseases of the Abdominal Viscera, when speaking of chronic obstructions of the liver, observes, " There is also very commonly a sensation of fluttering at the pit of the stomach, which I imagine arises from the blood of the vena portarum being, in like manner, unable to find a free passage; it is, therefore, retained in the vein, and thus causes a sensation of undulation. The pulsation in these affections is of too undulatory and diffused a nature to be mistaken for aneurism.

7. The *lungs*, in some diseases, are changed from their natural structure to a condensed substance, resembling very much the appearance of liver. When the lower and acute margins, where they lay over the pericardium and in contact with the diaphragm, are thus changed, they will give rise to pulsation in the epigastrium. The beating is observed by Mr. Burns, in a case of this kind which he witnessed, to have been as violent about the scrobiculus cordis, as from enlargement of the heart; and that it could be distinctly seen externally. The patient sunk under an increase of the disease of the lungs,—when they were found in the state above described.

8. Some disease or mal-conformation of the tricuspid valves, or a preternatural dilatation or enlargement of the heart in general, or of the right side only, are known to give rise to the same phenomenon. The pulsation in the epigastrium from an enlarged heart, will not be in perfect correspondence with the action of the heart itself; and when we recollect that it is not the heart itself we feel beating, but the left lobe of the liver driven suddenly forward by the violent action of the heart, we can understand why the beating of the heart and the pulsation should not be simultaneous.

9. Of all the causes of this kind of pulsation, an *adhesion of the heart to the pericardium*, it may be apprehended, is the most frequent. The pulsation, in these cases, is in perfect correspondence with the action of the heart. It is no way difficult to explain this fact, when we advert to the connexion which exists between the diaphragm and pericardium in a natural state, and when we consider that the latter, from disease, becomes firmly united to the heart, so that these parts all move together. The heart is now so encumbered by the pericardium, that this membrane no longer regulates its degree of distension; for it has actually become a part of it. Every contraction of the heart now pulls upon the diaphragm in such a manner, that, with the connexion of the liver to the diaphragm, the pulsation of the heart will be felt in the epigastrium, and will be synchronous with the action of the heart.

Dr. Mott concludes with observing, that the present essay is to be considered as an attempt to bring into notice some of the causes of an extraordinary and frequently anomalous affection, and must be viewed only as an outline, to be filled up by future observation and research.

*Case of Carotid Aneurism cured by an Operation;* by WRIGHT POST, M.D.

*Case of Brachial Aneurism cured by tying the Subclavian Artery above the Clavicle;* by the same.

The subject of the first case was a female, aged 32. The aneurism was of the size of a hen's egg. Two ligatures were applied on the trunk of the artery, three quarters of an inch asunder, and the vessel divided between them. Some degree of pain in the head, accompanied with vomiting, came on after the operation; which subsided in a few hours: no other unpleasant symptom ensued. She was discharged from the hospital in about a month after the operation: at this period, the tumor was little more than one-fourth the former size. An indistinct pulsation could be felt in it; but whether in the tumor itself, or whether it arose from the impulse of the surrounding vessels, could not be determined.

Several articles contained in this volume have been passed over unnoticed, from their containing matter, either principally of merely local interest, or of not sufficient novelty or practical utility to repay the attention of readers in the perusal of them. A very interesting and erudite paper, on the *Uniform Action of the Absorbent Vessels,* by Dr. C. E. DE PUY, must, however, be excepted from this class; but we purpose to insert an abridgment of this dissertation in our Collectanea, at some future period.

*Pathological Observations, illustrative of some Points in Physiology;* by F. LALLEMAND, M.D. &c.

(*Continued from p.* 441.)

M. LALLEMAND next proceeds to relate the results of his pathological researches on other subjects. Some interesting facts which occurred to his observation at the Hôtel-Dieu, gave origin to reflections on the *Functions of the Digestive Organs*, which contribute some useful additions to our physiological knowledge, and furnish hints for the improvement of the practice of medicine.

There was a woman in the Hôtel-Dieu, who, from fright and other causes of disturbance of the system during menstruation, had suffered a suppression of that secretion for six months previously to her admission. During that period, she had experienced an attack of hæmatemesis at the recurrence of each menstrual epoch, which continued three or four days.

When the process of digestion commenced, she experienced a slight rigor, accompanied with coldness of the extremities and a sense of heat about the epigastrium. After about half-an-hour had elapsed, the congestion of the stomach terminated in effusion of blood; of the occurrence of this she was previously sensible, from the cessation of the heat, and a sensation of weight and uneasiness about the stomach: this was soon followed by nausea and vomiting. It is a very extraordinary circumstance that she never threw up any thing but coagula of blood, which were sometimes considerable in quantity, and always without the least admixture of the food or drink which she had taken a short time previously. The vomiting generally ceased in about half an hour; she then became easy, and digestion continued as well as in the state of perfect health. M. Récamier, in his remarks on this case, cited many analogous facts, which seem to prove that the stomach has an elective action on the different substances which it may contain; and that, in the efforts of vomiting, it permits some to escape, while others are retained.

M. Lallemand next adduces an instance to prove that, in the efforts of vomiting, the action of the stomach may be so violent as to cause a rupture of that organ. A patient who had suffered from difficult digestion during five or six months, finding herself much better after the regimen that had been adopted, which was a very restricted course of diet, thinking to make amends for the privations she had endured, ate until she was fully satiated. She soon began to experience a sensation of weight about the stomach, with inclination to vomit; but the efforts she made to disembarrass her stomach were not attended with success. Suddenly, in the midst of extreme anguish, she experienced great pain in the abdomen, accompanied with a lacerating sensation: she uttered a piercing shriek, and fell down senseless; she became covered with cold sweat; the efforts to vomit ceased; the belly became more soft, although still much distended. After this, she for a short time appeared calm; but her state soon became more and more alarming, and she expired in the course of the ensuing night. On opening the body, the cavity of the peritoneum was found to contain a large quantity of half-digested food, of a pungent odour; the anterior and middle part of the stomach was lacerated obliquely from its small towards its large curvature, to the extent of five inches. The edges of the opening were thin and irregular, and the surrounding parts did not show any mark of previous disease. The three membranes of the stomach were not lacerated to the same extent, nor exactly in the same direction. The rupture of the peritoneum was greater than that of the muscular membrane; that of the mucous membrane was the least. It appeared as if they had been separated by dissection to the extent of an inch round the opening: this seemed to be owing to the different elasticity of these three tissues. The pylorus presented a circular contraction arising from scirrhous enlargement, an inch and a half in extent. The rest of the stomach was in a perfectly healthy state: the cardiac orifice was free, and had not undergone any change of structure.

The above-mentioned facts, observes the author, prove that the stomach performs an important part in the action of vomiting: we see that it will reject certain substances and yet retain others, on which it will act in the ordinary manner. If vomiting took place merely from mechanic causes, by simple pressure exerted on the surface of the stomach, we cannot conceive how this election could be made. It appears that the cardia performs the same office with respect to the action of vomiting, that the pylorus does to that of digestion. We cannot with precision compare a bladder adapted to the œsophagus, with a stomach possessed of vital functions. If the cardia contracts powerfully, vomiting will not occur, however violent may be the efforts to induce it: we have an incontestible proof of this in the case above related. It also shows us to what degree the actions of the stomach may be carried in this truly convulsive act.

M. Lallemand adduces several observations and judicious remarks, to show that vomiting cannot be attributed solely to the action of the diaphragm and abdominal muscles,—a notion adopted at the present period by some French physiologists of considerable talents, and which they have attempted to prove by experiments on brute animals. These opinions have not been favourably received in England; it will, therefore, be useless to notice the arguments brought forward to controvert them: we pass over these and some other reflections of minor importance, to the consideration of the subsequent section of this work.

The success obtained by M. Dupuytren, from his mode of treating cases of preternatural anus, the consequence of strangulated hernia, drew from all parts of France to the Hôtel-Dieu the unfortunate victims of this disease, and furnished M. Lallemand with opportunities for making some interesting observations *on Digestion*, and particularly *on the action of the stomach on different species of aliments.*

The cases to which we allude are admirably adapted for observation, and the deduction of physiological conclusions relative to man in a state of health. The nature of the chyle produced from different aliments,—the changes that take place in that fluid from its having traversed a greater or less extent of the small intestines,—and the means of repeating experiments on the same individual, are here offered to our view; and, what is of great importance, the stomach, in many instances, is apparently in an healthy state: the results of the exercise of its functions may, therefore, be considered similar to those which take place under natural and ordinary circumstances.

All the patients whom I have interrogated, observes M. Lallemand, have experienced a rapid emaciation during convalescence from the accident productive of this malady. This was particularly observed in the period more immediately subsequent to that event, and was accompanied with great diminution of strength, and an insatiable appetite; circumstances evidently dependent on the cessation of absorption from a part of the intestinal canal. The reparation of the system through the medium of this function being indispensible for the action of the organs most important to life, the activity of the absorbent vessels becomes increased, that they may obtain from other tissues of the animal economy materials which may supply the place of those no longer furnished by digestion. Thence arises emaciation and debility. We may well conceive, that, on a portion of the absorbent surface of the mucous membrane of the intestines no longer performing its functions, that the deficiency may, to a certain extent, be supplied by a more frequent renewal of the ingestion of aliments. This is what really happens in persons thus affected, who eat more frequently than persons under ordinary circumstances. The same thing may be observed after acute diseases and a prolonged spare diet. All the organs have occasion for reparatory materials; the whole animal economy suffers from the want of them: it is the stomach that demands them, because it is to that the substances should be confided which are to furnish to the blood the necessary fluid; and the painful sensation which leads to the intromission of food is more imperious as the necessity for it is more pressing: the stomach is hardly empty when a craving for food re-appears. It appears, then, that the sentiment of hunger depends more on the necessity of the supply of chyle to the blood, than on vacuity of the stomach.

Persons affected with preternatural anus, continues the author, do not supply the want of absorption from the lower part of the small intestines by this frequent use of food, since the emaciation of body continues to increase for a considerable length of time, and weakness of their moral qualities ensues, proportionate to that of their physical.

It, however, appears that the absorbents of the mucous membrane of the intestines, which preserve their functions, have their power gradually increased, according to a general law of the animal economy; for the emaciation ceases to make further progress after a certain length of time, and at the end of a few years those patients have, in general, acquired a state of *embonpoint*, with a tolerable degree of bodily strength; the appearance of the aliment evacuated has become different from that which it had in the earlier stages of the disease. These phenomena, it is evident, will vary according to the length of the portion of intestine superior to the artificial opening, and the temperament, age, and sex, of the patient.

All the patients thus affected, who came under the observation of M. Lallemand, had renounced the use of fruits, leguminous vegetables, and all aliments the basis of which is fecula. They observed that these aliments furnished them but little support, and appeased their appetite but for the instant: all, without exception, had been led by experience to eat only animal food. It was observed, that vegetables remained in the stomach for a much less length of time than animal substances,—generally only about half as long. When one patient, M—, had eaten animal food with a small proportion of bread, the digested aliment did not escape from the preternatural opening in the intestine until two hours had elapsed; vegetables, alone, were passed after one hour. Another patient, C—, retained the former about four hours; the latter, only two, or two hours and-a-half at the utmost: the other patients presented analogous results. In all of them, beans, peas, potatoes,

&c. although they had undergone culinary preparation, and been formed into a sort of pottage, escaped almost without having suffered any change. Crude fruits passed in small, compact portions, without having experienced the smallest alteration. Green vegetables were almost always immediately expelled, presenting their original colour and general appearance. Bread was retained for a long time, as well as meat boiled, but not so long as when roasted. The chymous fluid formed by the latter was more smooth and uniform than that of the former substances; and the articles of which it was composed could not be recognized.

The form and state in which the aliments were introduced, influenced the period of their retention. Thus, hard substances but slightly chewed, and the tissues of animal matter which contain a great proportion of gelatine, and of which the cohesion was not lessened by culinary preparation, were retained longer than the same substances under opposite circumstances.

But the cohesion of the aliments had not so much influence on the rapidity of digestion as might be supposed. Thus, for example, eggs in a soft or liquid form remained longer in the stomach and intestines than small pieces of apples or pears in a crude state; indeed, fruits that had undergone culinary preparation were less quickly voided than the same fruits in a crude state.

These facts were invariably observed in eleven patients who were in the Hôtel-Dieu; and several others, whom the author questioned at the Hospital of Invalids, stated that they had experienced the same results. They were derived from experiments made with a great variety of aliments, in order that general conclusions might be deduced from them.

On considering the above facts, the author continues to remark, we, in the first place, perceive that the aliments which remained the greatest length of time in the stomach and intestines were among those which have been always regarded as the most nourishing. Thus, as it has been long considered, the different species of food do not pass from the stomach in the order in which they may have entered it. It is not the substances that are most rapidly altered, or which offer the least resistance to the action of the digestive powers, that escape first; since those which soonest present themselves at the accidental opening in the intestine are precisely those which have the most nearly preserved their natural form and aspect, and possess the least nutritive qualities. (This does not depend on their state of solidity, or of softness or fluidity, since the same thing happens with crude fruits, spinage, &c. as with milk, &c.) The length of time, therefore, that aliments remain in the stomach is relative to the proportion of nutritive matter they contain, and to their state of animalization. As it is the pylorus that appears to retain them in the stomach, we must admit that it has an elective action, a peculiar sensibility; that it executes, as its name imports, *the office of a vigilant porter* (πυλωρος)—in this sense, that it obliges those substances to remain in the stomach on which that organ may act with profit for nutrition.

But, as it permits others to pass, more or less completely in the state in which they entered the stomach, we cannot say, with the greater number of physiologists, that it continues to reject their transmission until they have undergone suitable elaboration, permitting nothing to pass into the intestinal canal that has not been sufficiently altered by digestion: we would, on the contrary, state that it permits those substances to pass first which contain least alimentary matter, in whatever form they may present themselves, even though they have not suffered any alteration, (since we have seen them escape in the state in which they entered the stomach); whilst it retains the longest those which contain the greatest proportion of materials adapted to the functions of the stomach. The labour of the stomach is, then, proportionate to the quantity of nutritive matter contained in a given volume.

It is rather remarkable that those substances which have always been considered as heavy and indigestible, are in reality those which are the most nourishing: they are only indigestible for weak stomachs; and it is those which concentrate the vital powers in the stomach particularly, causing drowsiness and confusion of the mental faculties, as was well observed by Pythagoras, and is known by those who lead a sedentary life in cities, literary men, &c; but these are also the substances that are preferred by persons who work hard and take active bodily exercise, because they allay the sensation of hunger for the greatest length of time; and it is for this reason that pork is generally used by husbandmen and labourers. Hippocrates arranged these substances amongst those which he designated by the terms βαρὺς, and ἰσχὺρος, those which are heavy and offer resistance,—as beef, pork, eggs, &c.

It may, therefore, be admitted as an axiom, that digestion will require, on the part of the stomach, an exertion of its functions so much more prolonged, active, and energetic, as the matter to be acted on may contain, under a given volume, more nutritive particles; and these, we know, are animal substances, or those vegetables which in some degree approach to them by the nature of their composition. On the other hand, we have facts adduced to show that the substances the least nutritive,—that is, the least animalized,—are those which are with the most difficulty made to undergo the process of digestion, and at the same time those on which the digestive organs soonest cease to act. This explains an apparent contradiction, which would otherwise be with difficulty accounted for. We may observe, generally, that the more the composition of an alimentary substance is simple and different in its character from that of animal bodies, the more refractory is it to the operation of the stomach, and more difficultly transformed into our own substance, and *vice versâ.* This perfectly agrees with what was observed respecting the state of the alimentary matter on its escape from the accidental opening in the intestines; and yet the first are usually considered as aliments light and easy of digestion; while the latter, on the contrary, are regarded as indigestible.

M. Lallemand deduces the following conclusions from the above-mentioned facts:—

1st. That, if it be true that alimentary substances the most perfectly animalized contain the most nutritive matter, it does not thence follow that they are the most rapidly digested.

2dly. That, on the contrary, the process of digestion is more long and laborious as, in a given volume, the aliment contains more nutritive matter, and *vice versâ*.

3dly. That the aliments do not escape from the stomach in the order in which they are introduced, but that it is not those which are first altered by digestion that pass the first: it is those, on the contrary, which, containing least alimentary matter, are most refractory to the digestive powers.

M. Lallemand next directs his views to *the action of the intestines;* some reflections respecting which are furnished by the contemplation of the above-mentioned facts.

We have already noticed that many substances escaped from the accidental opening in the intestine nearly in the same state as that in which they were introduced into the stomach: we must also observe that the same substances were more or less altered in different patients, according as they were retained for a longer or shorter period; and that all the concomitant circumstances tended to show that this difference depended on the greater or less length of the portion of intestine between the stomach and the wound. These substances, we may likewise observe, can hardly ever be recognized on their evacuation, in persons in a state of health, in whom they have traversed the whole course of the intestinal canal. M. Lallemand thence supposes, that the process of digestion is not exclusively confined to the stomach, but that it continues throughout the whole of the intestines—(we apprehend he means only the small intestines); and that the functions of the latter are not merely occupied in the separation and absorption of the chyle from the chymous mass of alimentary materials. This, he observes, was also the opinion of M. Gosse, who was led to form it from experiments made on himself.

Those patients, in whom the whole of the residuum of the alimentary matters escaped by the wound, passed by the anus, every fifth or sixth month, a large hard mass of a greyish colour; which was nothing else than the mucous matter secreted by the intestines between the wound and the anus, which had gradually collected and become hardened in the rectum; which proves that the mucous membrane of the intestines continues the process of secretion, although the particular functions of those organs have ceased. This collection of fecal matter is compared to the meconium of the fœtus.

We now arrive at the conclusion of this work; and, although it has already engaged more of our time and attention than we are often disposed to devote to ponderous volumes, yet we drop the pen with reluctance on being obliged to close our conference with M. Lallemand; so much have we been gratified with the contemplation of the facts with which he has here furnished us, and his judicious and original reflections respecting them: but the traits of an ardent and comprehensive genius, zealously devoted to the improvement of medical science, so strongly depicted in this work, lead us to hope that this is only a precursor of still more valuable additions to our knowledge from the same source. We must not, however, conclude, without adducing the following general reflections of the author:—

"On considering in succession all the organs of the animal economy, it would be easy to show that there is not one of them respecting the functions of which medicine and surgery will not furnish as many positive facts, directly applicable to man, as may be deduced by analogy from zoology and the dissection of living animals. We may perceive that the different tissues which enter into their composition are not less distinct in their diseases than in their physical characters; and that, even in this respect, pathology has anticipated physiology, (M. Pinel had arranged the diseases of the different mucous structures, &c. before Bichât published his treatise on those membranes), and has constantly furnished it with its most important materials. It is to the admirable manner in which Bichât has applied this knowledge, that we must attribute the charm and interest spread over his immortal works. We may perceive that it is from the study of medicine that we must acquire a just idea of the animal economy, taken in its whole extent, of that vital power (φυσις, ενορμον) distributed throughout its whole structure. It was by the study of medicine that Hippocrates arrived at the discovery of those eternal fundamental truths, which are not less applicable to man in the state of health than in a state of disease."

Some of those aphorisms of the venerable sage of Cos terminate the work of M. Lallemand; to whom the sentiments uttered by the great physical poet, on another occasion, appear correctly applicable:—

> "Te sequor, ô Graiæ gentis decus, inque tuis nunc
> Ficta pedum pono pressis vestigia signis,
> Non ita certandi cupidus, quàm propter amorem
> Quod de imitare aveo."

---

### *Case of spontaneous Disunion of a recently-united Fracture of the Humerus.*

This case is related by Prof. Hufeland,* who attributes the ill consequences attending it to the effects of the waters of *Carlsbad.* Although we are disposed to doubt of the correctness of this explanation, and to consider the case in another point of view, yet, the fact itself being interesting, we shall give a brief relation of the principal circumstances attending it.

---

* Journal der Praktischen Heilkunde; Oct. 1816.

M. de T— fractured the os humeri of the left arm. Fifteen days afterwards it was considered to be firmly united, and he was permitted to go to the waters of Carlsbad; the use of which have been advised for some abdominal affections. On his arrival at Carlsbad, the patient consulted Dr. Brieske, who considered that the fracture had been properly treated, and was doing well. Dr. B. directed the use of bandages, &c. to be continued. He took the waters, without experiencing any particular effects from them on the stomach and bowels; but he began to suffer darting pains in the situation of the fracture: when this was examined, the uniting medium was found to have become soft. The use of the waters was persevered in. The pains increased in severity; and, when the fracture was again examined, (sixteen days after having commenced the use of the waters,) the callus had entirely disappeared.

### *On the use of Moxa in Hydrocephalus.*

M. Regnault has lately published a Memorial on Hydrocephalus, with the intention of making known the beneficial effects he has derived from the use of moxa in the treatment of that disease.

The author first traces a sketch of the history of the disease, and the pathological opinions which have been adopted respecting it at different periods, and makes some judicious remarks on its real character, and the appropriate mode of treatment in its early stages; but we shall confine ourselves to the relation of those which apply to the use of the remedy to which we have alluded.

"The process consists," observes M. Regnault, "in the application of successive trains of moxa, or little rolls of cotton, in two lines; the direction of one of which should be from the middle of the forehead to the eminence of the occiput; the other, from one temple to that on the opposite side. The rolls should be but slightly twisted, so that the combustion may not be too active, and the emission of caloric too abundant: they may be of about four or five lines in diameter, and eight or ten in length, regulated according to the age of the patient and the presumed thickness of the integuments. After having determined on the situation in which it is to be applied, the part is to be covered with a strip of strong close-grained woollen cloth, about three inches in diameter, on which the coil of moxa is to be placed;* a stream of air may then be directed on it by appropriate means, sufficient to maintain combustion. Moxa, applied with these precautions, does not produce actual cauterization, but a considerable degree of redness and slight tumefaction of the part, which becomes covered with drops of limpid serum.

"It remains for me to point out the cases where this measure may be productive of injury," continues the author, "and those in which it may be employed with hope of success.

* It may be confined in its situation by a tube of pasteboard, or covering the cloth with the white of an egg.

"It is evident, that, whenever there is severe pain about the temples, redness of the face and of the conjunctive membrane of the eyes, acute fever, great agitation,—indeed, any of the signs of increased afflux of blood to the head; that a measure like this, adapted to increase that occurrence, should not be employed. But, when those symptoms have disappeared, and those alone remain which announce compression of the brain, or dilatation of it from the presence of fluid within the ventricles; there is then no reason to prevent the application of moxa, with the precautions above indicated.

"The use of this remedy should not make us dispense with purgatives, and other measures that may be considered adviseable; for, I repeat, I have wished to point out a method of treatment which I believe to be useful, not to hold forth a specific.

"I shall now relate two cases in support of the principles I have advanced in this memorial, and which, I hope, will confirm the propriety of them.

"An infant, aged eighteen months, of a well-marked lymphatic temperament, was brought to me in the year 1808. This infant, pale, and tolerably plump about the superior half of the body, fell into violent convulsions whenever it was forcibly shaken. Excepting under these circumstances, its inferior extremities, which had for a long time been in a state of marasmus, were never observed to perform spontaneous motion. I remarked, in the first place, a striking disproportion of the size of the cranium to that of the face,—a disproportion which showed the existence of hydrocephalus in a very advanced stage. The cavity, particularly about the posterior part, was of nearly double the ordinary extent. The sutures and fontanels were very large, and a fluctuation could be felt through the interosseous membrane; the bones were hardly compact, and gave way to a slight impression of force; and the eyes projected so as to be nearly without the orbits. The digestive organs were weak, the tongue pale, and obstinate costiveness announced the torpid state of the intestines; the appetite was almost destroyed. The whole of these symptoms, some of which appeared during the first month after birth, would not permit me to hesitate an instant in forming an opinion on the nature of the disease. I directed animal broths, a small quantity of wine, and the use of bitter and steel medicines. Twice a week, the patient took one or two grains of calomel; and gentle purgative glysters were frequently administered; and every third or fourth day the moxa was applied to the cranium. After it had been employed four or five times, the convulsions were less readily excited; they after this gradually ceased, and the infant might be shaken with impunity. Nutrition became better effected; the inferior extremities recovered their plumpness and natural motion. After this mode of treatment had been continued three months, the volume of the head was evidently diminished; the bones more nearly approached each oth^r, and were less flexible; the eyes were less projecting; the constipation of the bowels disappeared; and I had the satisfaction to consider that I had snatched this infant from almost inevitable death. (The moxa was applied eighteen times.) I have seen this patient since that period: the bones have acquired the necessary solidity; the head is still a little voluminous, but health is perfectly established.

"The child of William Clark, aged thirty months, born of scrofulous parents, presented the following symptoms when I was consulted respecting it:—an extraordinary bulk of the head, and relative smallness of the face; a tumor, slightly projecting, along the course of the sagittal suture, in which there was sensible fluctuation; an habitual hanging-down of the superior eye-lids; projection of the eyes, with a continual flow of tears; the commencement of blindness; an invincible repugnance to motion; frequent moaning; disturbed sleep; voracious appetite; habitual salivation; alternation of diarrhœa and constipation; great general emaciation, particularly of the inferior extremities, which were constantly half bent, and nearly paralyzed; slow pulse; and frequent recurrence of convulsions, particularly on the slightest motion. Without expecting much success in so desperate a case, I directed a nutritive diet, and small doses of cinchona wine and other bitters, when the state of the abdominal viscera permitted them; a gentle purgative was cautiously exhibited from time to time. Twenty-two applications of the moxa were successively employed, in the manner already directed. A continuance of this mode of treatment during six months, carefully and methodically conducted, was sufficient to remove this alarming train of symptoms: the digestive functions were re-established; the cranium gradually, though slowly, recovered the natural size; the commencement of amaurosis disappeared in the same manner, as well as the convulsions; and at length health was completely restored. The solidity of the bones also, after a considerable length of time, has become effected.

"Although the moxa was not employed alone in the treatment of those two cases, I do not hesitate to attribute, in a great measure, the remarkable success I obtained to that remedy; for I have employed purgatives alone without any sensible benefit in numerous instances.

"These facts evidently demonstrate—1°. That the application of moxa, in a gentle manner, may be made without inconvenience on the head of the youngest infants affected with chronic hydrocephalus.

"2°. That this measure eminently contributes to the success of the treatment, and that it should not be omitted in any instance where there are no symptoms present which contra-indicate its use; such are those which make us presume that a great degree of cerebral irritation exists.

"Should it be asked, how the mode of action of moxa may be explained, I would reply, without attaching much importance to the investigation, that the local irritation which it produces solicits towards the external parts, the fluids which had previously been determined to the organs within the cranium,—thus changing the morbid direction of the vital actions, preventing further exhalation of serum, and favouring the removal by the absorbents of that which had already been effused. The purgatives contributed much to the production of this object, by establishing in the intestinal canal a supplementary secretion; while the aliments exhibited furnished the materials for nutrition, which was favoured by the use of tonic medicines.

"Such are the reflections which have been suggested from observation and experience: that they may engage the attention of practitioners, and encourage them to employ a measure from which no ill effects can be feared, is what I desire. I am far from exaggerating the utility of it, but it appears to me that it will contribute to arrest the progress, and prevent the fatal termination, of a disease generally considered as incurable."

M. Esquirol, a short time since, read a Memoir to the Medical Society of Paris, containing the result of his observations at the *Salpêtrière*, during the years 1811, 1812, 1813, and 1814.

The number of insane persons admitted into that hospital during this period was 1,119; ninety-two of whom became insane after child-birth, during lactation, or at the time of ceasing to give suck.

Insanity manifested itself from the first to the fourth day subsequent to delivery, in 16 women;

From the fifth to the fifteenth, in 21;

From the sixteenth to the sixtieth, in 17;

From the sixty-first to within a year, in 19;

Immediately after ceasing to give suck, either voluntarily or forced, in 19.

The development of the malady is consequently more to be feared in women recently delivered, than in those who have given suck for some time; and it becomes more rare as this period is extended.

Of these 92 insane persons, 8 were in a state of folly, 35 in that of melancholy, and 49 were maniacs;—22 were from 20 to 25 years of age; 41 from 25 to 30; 16 from 30 to 35; 12 from 35 to 40; 2 were above 40 years old;—63 were married, 29 unmarried women.—14 became deranged from the action of physical causes, (almost all of them from the impression of cold;) the remaining 78, from moral causes.

Of 55 who recovered, 4 did so during the first month; 7, during the second; 6, during the third; 7, during the fourth; 5, during the fifth; 9, during the sixth; 15, from the sixth to within two years; and 2, after two years.

Of the 37 who were not cured, only six died. Examination of the body after death did not afford any information respecting the cause of the disease.

A singular pathological phenomenon has been noticed by M. Esquirol, in the bodies of many lunatics, after death—the displacement of the transverse portion of the colon; on which he makes the following observations:—

"The ancients and the moderns who have treated of mental alienation, and particularly of melancholy, have all spoken of lesions of the abdominal viscera; but no author has mentioned a displacement of the transverse portion of the colon. A displacement of this intestine may, however, be frequently observed in the bodies of insane persons. Sometimes, the direction of it is oblique; at others, perpendicular, so that its left extremity is situated behind the os pubis.

"This displacement cannot be attributed to mechanic action, dependant on thickening of the coats of the intestine, or a collection of fæces in its cavity; for, in the greater number of subjects that I have examined, the colon was empty, and in all it was in a healthy state as to structure. The same was the case with the ascending and descending portions of it, which, by their relative situation, could draw it from its natural direction. This displacement is not the effect of the last disorder under which the patients die; because this circumstance has been observed in those lunatics who have died of different diseases.

"The insane persons, particularly melancholics, in whom this displacement was observed, frequently complained of pains in the epigastric region, which they described as feeling as though a cord were tied round the body about the hypochondria, and the stools were generally in a bad state. May not these symptoms be explained by the displacement of the colon?

"Have not the ancients, in administering hellebore; and the moderns, in prescribing emetics and drastics, in the treatment of mental alienation, particularly in melancholy; aimed at restoring the healthy state of the abdominal viscera? But, may not purgatives be considered injurious, since they increase debility of those parts? and, thus, have they not taken care to join them with tonics? Lastly, do not sea-voyages, and horse-exercise, so beneficial in melancholy, act by strengthening the abdominal viscera particularly.

"The knowledge of these facts has appeared interesting to me,—1st, because this displacement is frequent in insane persons, particularly in melancholics; 2dly, because an acquaintance with this fact may lead to a more decided and rational mode of treatment in that malady."

---

THE following case occurred a short time since to the observation of Dr. DUPLAN, of Tarbes; and is related in the *Journal Universel des Sciences Medicales*, by Dr. FOURNIER.

On the 31st of March, 1818, Dr. Duplan was requested to see a lady, who had suffered from strangulated femoral hernia for ten days previously. The surgeon, who had attended her, had bled her daily for nine days; after which, he deserted her. Dr. D. on his arrival, found her labouring under great anxiety: her pulse was small and quick, skin dry, and countenance much changed. He found a cataplasm applied to the hernia, which was of the size of a goose's egg, soft, and fluctuating, and of a brown colour on the surface, except about its base, where it was of a purplish hue. The cellular membrane surrounding it was hard and unyielding. On the exterior edge of the tumor there was a papular projection, the skin of which was so thin and disorganized that it was ruptured by slight pressure, and about two ounces of pus, of a feculent odour, escaped from the opening. The intestine was exposed, which was found to be a fold of the small intestines, of about three inches in length; the opening through which the feculent matter had escaped was about four lines in diameter. The edges of the opening, to the extent of two or three lines, was of a dark-gray colour, and disorganized in texture. The rest of the intestine was much inflamed.

The formation of an artificial anus appearing to be the only means by which the patient could expect to survive from this accident, Dr. Duplan passed a waxed thread through the mesentery, and then removed, with a scalpel, the whole of the mortified part of the intestine. The two ends of the intestine were then brought as nearly as possible into contact, and, by means of the waxed thread, maintained in adherence with the external wound. The patient was desired to lie quietly on her back; a small quantity of boiled meat was given her, an enema injected, and veal-broth was allowed as common drink.

Although she took no other nourishment than a small portion of boiled meat, and an ounce of wine every fourth hour, the quantity of feculent matter evacuated from the wound was very great for the first twelve days after the operation, and the dressings were obliged to be renewed seven or eight times in twenty-four hours. During this time, several *lumbrici* escaped from the wound. From this period, the quantity of feculent matter evacuated began to diminish; which may be attributed to the strict diet of solid food to which the patient was confined. The wound began to look clean on the 6th of April (the seventeenth day from the operation), and became perfectly healthy in appearance about the 17th of the same month. Granulations then rose on its whole surface, and the extent of it became sensibly diminished. On considering the favourable appearance of the wound, Dr. Duplan felt assured that the intestine had contracted adhesion with the border of the crural ring, and that the thread had therefore become useless: it was consequently removed.

Enemas, alternately emollient and slightly purgative, were injected daily. Until the 15th of April, they were voided unaltered in appearance; at this time they had a pale yellow colour when returned: those of the 16th, 17th, and 18th, were mixed with a small quantity of whitish mucus; those of the 19th, 20th, 21st, and 22d, were similar to those of the 15th. On the 23d, at the time the enema was administered, the patient voided a small quantity of stercoraceous matter, of a firm consistence; and from that time the fæces continued to pass in the natural way.

After the fifteenth day from the operation, merely a small quantity of greenish-coloured mucus, of a slight fœculent odour, escaped from the wound. From the twenty-second to the thirtieth day, the lint covering the wound was merely moistened with healthy inodorous pus; and the wound was completely cicatrized on the forty-sixth day from the operation.

The diet was confined to four ounces of boiled meat, and an ounce of wine, every four hours: in the intervals, the patient took four ounces of decoction of cinchona with six ounces of mint-water; her strength was very soon much improved, the pulse became full, the dryness of the skin gave place to a slight moisture, and her countenance assumed its former appearance.

---

*A Mode of extracting Stone from the Bladder* has been employed with success, in one instance, by Dr. Calvin Conant, of the United States, which merits universal attention. The patient was a youth, 15 years of age, who had for a long time suffered frequent suppression of urine, in consequence of a loose stone which fell into or upon the sphincter of the bladder. Dr. C. furnished himself with a very fine silver-wire, about twenty inches long, made elastic by being frequently drawn through a wire-plate without being annealed. He then drilled two holes through the point of the catheter, upon the convex part, about one eighth of an inch asunder, through which the ends of the wire were passed, bringing them out at the end of the catheter. The ends of the wire were graduated into spaces of a quarter of an inch each; by which he could judge of the size, and in some degree of the form, of the stone. Having introduced the catheter, and enlooped the stone, which was done with little difficulty, he examined the scale, and found one inch and a quarter of the wire was taken up in surrounding it. The situation of it was changed until it was found that it was enclosed in such a manner as to occupy the greatest length of loop, which was one inch and seven-eighths. Having drawn the ends of the wire very tight, to keep the stone firmly enclosed in the loop, Dr. C. proceeded, with gentle varied motion, to draw it through theurethra; which was effected with but little difficulty or pain. No unpleasant circumstances followed the operation.

---

